# Organizational and Systems-Based Framework to Guide a Departmental Approach to Academic and Leadership Advancement for Women in Emergency Medicine

**DOI:** 10.1016/j.acepjo.2026.100394

**Published:** 2026-04-17

**Authors:** Kori S. Zachrison, Mariam Kapanadze, Dara James, Nour Al Jalbout, Rebecca E. Cash, Wee-Jhong Chua, Valerie Dobiesz, Daniel J. Egan, Erica L. Nelson, Phuong Pham, Elizabeth Temin, Margaret E. Samuels-Kalow

**Affiliations:** 1 Mass General Brigham Department of Emergency Medicine, Boston, Massachusetts, USA; 2 Harvard Medical School, Boston, Massachusetts, USA

**Keywords:** women in emergency medicine, gender equity, professional development, gender disparities

## Abstract

**Objectives:**

To address gender disparities in career advancement in academic emergency medicine, we sought to identify specific and actionable approaches that an academic department could develop or enhance to create a sustainable pathway for advancement of women in academic and leadership roles.

**Methods:**

This project took place in an urban academic department of emergency medicine and included 3 phases: (1) a literature review to assess the current landscape, (2) interviews with key informants, and (3) a consensus process to organize and prioritize strategies.

**Results:**

Our literature review identified 55 manuscripts and key themes of mentorship, training, grant success, data and benchmarks, recognition, and institutional policies. Across 20 interviews with internal and external faculty members, themes that emerged were programs and training, barriers, facilitators, mentorship, and individual and system components. During our consensus review, potential strategies were organized by relative feasibility and importance. Ultimately, strategies for building standardized systems were prioritized for their achievability based on available resources and alignment with department priorities and policy.

**Conclusion:**

There are several strategic options that academic departments may pursue to address gender equity challenges. This includes building standardized systems to support equitable advancement of all faculty members across domains, including salary, support, and opportunities. Standardized approaches ensure that departments not only promote gender equity but also facilitate the professional growth and promotion of all faculty members.

**The Bottom Line**

This 3-staged project identified actionable strategies to address barriers to the advancement and leadership of women in academic emergency medicine. Through literature review, key informant interviews, and a consensus review, a framework was developed that may serve as a guide for promoting gender equity in an academic department of emergency medicine. A key cross-cutting theme is in the value of standardized systems that support all faculty, including in the domains of salary, support, and opportunity.

## 1 Introduction

### 1.1 Background

Gender disparities in career opportunities and advancement are well-documented and persistent in academic medicine,^1^, ^2^, ^3^, ^4^, ^5^, ^6^ affecting various domains including compensation, professional rank, retention, and leadership roles. These differences are often compounded. For example, women receive fewer invitations to speaking engagements such as major conference platforms or visiting professorships.^2^ Medical schools and departments use such talks as a measure of influence and impact in evaluations and considerations for promotion and advancement, thus further widening disparities. In this context, it is not surprising that, even after accounting for experience and academic productivity, men attain full professor status and leadership positions at higher rates.^5^

### 1.2 Importance

Despite growing awareness, there is still a substantial need for progress in addressing and mitigating these gender disparities. Although investment in targeted leadership development programs has increased, the field of emergency medicine saw no improvement in gender or racial/ethnic disparities in leadership and academic rank over the 6-year timeframe from 2015 to 2020.^7^ Existing systems and structures often include entrenched barriers, including organizational cultures and practices that disadvantage women.^1^ A growing body of literature has elucidated many related factors, such as under-sponsorship and the potentially exclusionary impact of informal networking practices.^1^^,^^6^^,^^8^^,^^9^ Although understanding barriers is critical to inform solutions, less research has been done to specifically identify strategies and solutions, particularly in the context of an academic department of emergency medicine.

### 1.3 Goals of This Investigation

Thus, we sought to assess the current landscape, to identify actionable approaches, and to propose programs that an academic department could implement or enhance to create a focused and sustainable pathway for women in academic and leadership roles. Our goal was to develop a “roadmap” that could guide work in our own department and serve as a reference for others as well. We also aimed to include considerations of practicality and feasibility in our final product. Overall, we looked to identify strategies that would both remove existing barriers and, even more, empower women to thrive, ensuring their contributions are recognized and their leadership potential fully realized throughout their careers. During the course of this project, many academic medical centers removed workplace programs targeting particular demographic groups.^10^^,^^11^ To address this potential concern, we developed recommendations that might be applied both at baseline and one where the programs and activities could not target a specific demographic group.

## 2 Methods

### 2.1 Study Setting and Overview

The study setting was an academic department of emergency medicine in the Northeastern United States. This department is affiliated with a medical school and has an established emergency medicine residency.

This 3-phased project included a narrative literature review, interviews with key informants, and a consensus process to organize and prioritize strategies. The work was led by a senior woman, an academic faculty member in an academic department of emergency medicine, with a committee of faculty and staff representing varied identity groups, levels of advancement, and degrees of clinical and research engagement.

### 2.2 Literature Review

We conducted a narrative literature review focused on identifying approaches and programs that a department could implement to advance women in academic and leadership positions. The search strategy was developed with the support of a research librarian. The search was conducted on July 9, 2024, and the terms are included as Supplemental Material I. All titles and abstracts were reviewed by 1 of 2 members of our team, and a senior team member adjudicated the final list of included articles. All included manuscripts were then fully reviewed by an additional team member, with identified themes extracted and entered into a spreadsheet for tracking.

### 2.3 Key Informant Interviews

From December 2024 to February 2025, open-ended qualitative interviews were conducted with key informants under the supervision of a team member (MSK) with advanced training, experience, and publications in qualitative research. Potential participants were identified by members of the committee and included female faculty working in the field of medicine from both within and outside of emergency medicine and the local institution; apart from gender, no other exclusion criteria were applied. Female faculty were intentionally selected as key informants because the study focuses specifically on issues related to female faculty advancement, making their perspectives most directly relevant to the aims of the work. Potential participants were first contacted via email with an invitation to participate in the 1-time interview. The open-ended questions were informed by the literature review and developed *a priori* by the research team to guide an in-depth, semistructured qualitative interview between the study staff and each participant. All interviews (∼30 minutes) were conducted by 1 member of the team (MK) with a master’s degree and specific training in qualitative interviewing and who did not have a previously established relationship with the participants. All interviews were conducted via Microsoft Teams or by phone, based on the informant’s preference. Verbal consent was obtained at the beginning of each interview. Interviews were audio recorded and then transcribed, with transcriptions securely saved and accessible only to the study team.

Transcripts were deidentified, anonymized, and coded for identification of key themes and subthemes. Two members of the study team (DJ, MK), both with qualitative analysis training, independently coded each of the 20 interviews. Through an iterative process, the team members compared coded versions of the interviews and subsequently developed a consolidated codebook with codes organized into themes and subthemes. Respective of the data, the study team selected quotations that represented and illustrated the key themes and findings. The guiding research question for analyzing the key informant interview transcripts was: “How can the department develop specific approaches and programs to recruit, retain and advance women in academic careers while building a pipeline of women leaders in emergency medicine?”

### 2.4 Consensus Process

All committee members reviewed the results of the narrative literature review and the themes and subthemes from the analyzed interviews. Using a final in-person meeting, they categorized the strategies identified through key informant interviews based on their importance and feasibility. A list of high-importance, actionable strategies—with an accompanying measurement plan—was developed for the department to implement.

The project was determined to be “not human subjects research” by our local IRB.

## 3 Results

### 3.1 Literature Review

From July 9, 2024, to September 10, 2024, we reviewed 114 manuscripts, of which 55 were identified for inclusion (Table S1). Publications ranged from 1992 to 2024.

Key themes that emerged from the literature review that were determined to be salient by the committee included: mentorship, training, grant application success, data and benchmarks, recognition, and institutional policies.

Literature review theme 1: Mentorship. Multiple studies reported significant challenges in finding appropriate mentors within emergency medicine; when found, characteristics of successful mentors included altruistic, approachable, available, and strong advocates for their mentees.^12^^,^^13^

Literature review theme 2: Training. These articles recommended training focused on specific career development skills, improved early career research training, and work-life balance and time management.^14^, ^15^, ^16^, ^17^, ^18^

Literature review theme 3: Grant success. Identified barriers to grant funding included (1) finding appropriate mentorship; (2) having appropriate time to prepare and submit a career development award; and (3) lacking a robust administrative infrastructure to support NIH awards. One identified strategy for improving future success was to have better access to small/pilot grants.^19^, ^20^, ^21^, ^22^

Literature review theme 4: Data and benchmarks. These articles provide data on ongoing disparities and highlight the impact of intersectional discrimination throughout the academic career path. Identified strategies included collecting baseline data, conducting ongoing evaluation, holding leadership (division chiefs, chairs) accountable, and developing a leadership position specifically focused on gender equity.^23^^,^^24^

Literature review theme 5: Recognition. Opportunities to improve equity in recognition were identified and included authorship (particularly senior authorship), promotion, and advancement. Strategies in this domain included encouraging women researchers to be primary authors for published research, supporting women in identifying available grant opportunities for their work, providing administrative support, and incentivizing and supporting women faculty to apply for awards.^25^, ^26^, ^27^, ^28^, ^29^

Literature review theme 6: Institutional policies. These were repeatedly described as playing a critical role and included sufficient parental leave, lactation support, salary equity, safety and harassment protections, financial support and sustainability of gender equity initiatives, and flexible options such as job sharing.^30^, ^31^, ^32^, ^33^, ^34^, ^35^

### 3.2 Interviews with Key Informants

We conducted 20 qualitative interviews with faculty members, including 13 from within our health system and 7 external faculty. We identified 5 primary themes with 14 subthemes. Representative quotes from interviews are organized by theme and subtheme (Table 1).

Interviews theme 1: Programs and trainings. We identified several areas of unmet need related to organized activities to support academic development, including the importance of optimizing measurement and evaluation, and initiating both specific and general programs. While recognizing the general value, 1 interviewee emphasized the importance of personalization, stating “it depends on the faculty member and what they’re interested in… [programs] not only provide the faculty with …content knowledge,… but… it’s an opportunity [to] network around our campus as well.”

Interviews theme 2: Barriers. A number of obstacles that hindered progress in academic leadership were identified, with subthemes including lack of knowledge and awareness of available departmental resources and/or the department and lack of transparency and trust. One respondent noted “we asked for deidentified data… we get a response that was like, well, we’ve reviewed it and it’s equitable, but [we do] not actually ever see the data.”

Interviews theme 3: Facilitators. Respondents emphasized the presence of opportunities that enable growth, including guidance on available opportunities and peer support. One described her practice that upon seeing an opportunity come across her desk, she asked herself, “who are three women I could forward this to.”

Interviews theme 4: Mentorship. This encompassed the advancement, guidance, and skill development that mentors provide to mentees, with subthemes such as support and connection, the importance of career trajectory mapping, and assistance with promotion. With respect to career trajectory, 1 respondent noted “when I came in, it took me many years to actually understand what I needed to get promoted… so I probably lost 5 years of just not really understanding.”

Interviews theme 5: Individual and systemic components. This theme included factors contributing to personal and professional well-being, with subthemes covering engagement and belonging, work/life balance, and the underlying policies and guidelines that enable faculty to be successful within a department. This theme also included administrative support for the promotion process, separating issues of gender and parenting in policy development, and implementation of policies that provided support for individuals at moments of transition (eg, new parents). As 1 respondent said, “I think there’s often a conflation of the advancement of women with the issues of being like a new parent or new mom. I’d love to kind of acknowledge that these are related but are not the same thing…Again, I think of them as floor level issues like we should just have them solved. They’re not going to solve advancement issues.”

**Table 1:** Representative quotes from key informant interviews by theme and subtheme.

Theme	Subtheme	Representative quote
Programs and trainings	Wants and needs	The first one is self-promotion and …communicating your value. I think there's a lot of gendered ways in which we think of ourselves, and women learn to communicate…I think a second one is finance. I think most physicians don't learn about finance nearly enough. But again, I think there's a bit of a bias that that often has been an obstacle to…leadership of not having comfort in that area.
	Measurement/evaluation	Let's say take all the senior roles and see how many women are there, but I think it's more about what it takes to get there and also who is left out.
	Specific programs	The Association of American Medical Colleges both early career and midcareer women seminars I think are very good for folks. I just recently finished Executive Leadership in Academic Medicine out of Drexel, which is probably the most well-known and respected women's leadership development course…I'm sure each individual institution has some internal programming…in emergency medicine…SAEM offers the chairs development program, and other programming depending on where you are sort of in your stage of career.
	General programs	I think it depends on the faculty member and what they're interested in and like I said we do have these leadership and management courses …it's great for people who might have ….an assistant director type of role, whether it's an associate program director or assistant medical director…those have been really popular and successful…They not only provide the faculty with you know, content knowledge,…but… it's an opportunity network around our campus as well.
Barriers	Lack of transparency/trust	For many, many years, you know we asked for deidentified data just to look at gender gaps and, you know, we get a response that was like, well, we've reviewed it and it's equitable, but not actually ever see the data…And I think that when there's not a ton of trust there at baseline or there's concern that there could be disparities when you're not actually having data shared with you…It leaves people feeling like, can you really trust what's being said…
	Lack of knowledge/awareness	I think especially as a junior faculty member like, you have no idea what you don't know, and you don't know what to ask people. And so I think even just like getting that started would be really helpful.
Facilitators	Guidance for opportunities	I think just having a centralized list of all these opportunities. And then giving someone a point person in your department who always highlights. These are opportunities and kind of taps someone on the shoulder periodically this is open to you.
	Peer support	She says that every time a job opportunity comes across her desk…She asked herself, who are three women I could forward this to, and so like, it's a very simple thing. But I started doing that ever since… many times women aren't going to think that they're eligible for a job as much as a man.
Mentorship	Support and connection	Most women have mentors. Their mentors may not be as powerful, and that leads to less opportunities. Anyway, it's sort of thinking about, you know, just having a mentoring program isn't enough, and that that sponsorship piece, just as I was talking about, like thinking about external leadership positions and how you can put people forward.
	Career trajectory/map	I'm talking specifically about academic promotion… when I came in it took me many years to actually understand what I needed to do to get promoted…So I probably lost 5 years of just not really understanding. So I think that if you can start that process when people first get here… look, these are the things that are going to be expected to go to the next step, here are ways in which you can start doing that.
	Promotion	I think it's 10 hours of administrative support where someone, an administrator, will go and do like the work of updating their CV, and then we're doing group coaching to help like keep them on track toward their promotion, like getting their materials together. What are your next steps? What's your motivation for this? Making sure you have all the materials that you need to get promoted. So that's another new initiative that we're about to start that is basically the idea of like taking a lot of the work out of getting promoted and your career so that you can focus on the actual work you are doing to make that happen.
Individual and systematic	Engagement and belonging	I certainly spend a lot of my time mentoring women around negotiation. But in terms of really fixing the problem, it's got to come from organizational systems-based change. And what's an example of this? Again using pay equity. So there is an increasing conversation nationally around working hard to eliminate negotiation for first jobs of junior faculty coming right out of training because there is a lot. There is very robust evidence that even in that setting…you have people coming right out of the same training programs and men and women, there's a pay disparity. So whose fault is that? Whose problem is that? …That's not an individual issue, that is a systems issue and so looking at standardizing offers for folks coming right out of training to kind of take that negotiation penalty off the table.
	Work/life balance	I just feel like this needs to be on the top priority of institutional leadership, either through faculty development or the Dean’s office…strategies to be able to…help with co- recruitment, for example. And then there are perhaps different funding opportunities to provide you know child support, for example, somebody wants to go to conference, but you know how about providing some child support so that they can travel either with their children or get some help at home.
	Policy and guidelines	I think there is often a conflation of the advancement of women with the issues of being like a new parent or a new mom. I'd love to kind of acknowledge that these are related but are not the same thing. So like a lot of the issues related to being a pregnant person or a new parent, I think are really important. Again, I think of them as floor level issues like we should just have them solved. They are not going to solve the advancement issues.

### 3.3 Consensus Review

Strategies identified through the literature review, key informant interviews, and committee discussion were reviewed and categorized by the committee based on their importance and feasibility (Fig).

**Figure:**
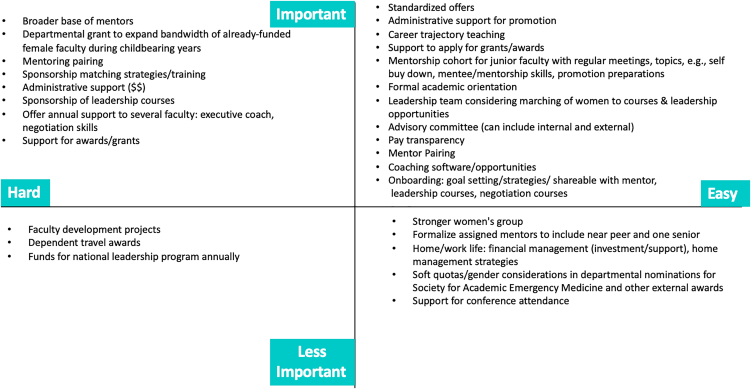
Committee recommendation ranking.

Highest priority recommendations (ie, important and feasible):

1. Educational initiatives: To strengthen faculty development, the committee recommended establishing structured mentorship cohorts tailored to career stage (eg, new and midcareer faculty), instituting a formal academic orientation for all incoming faculty, and delivering explicit instruction on career trajectories and promotion requirements. Additional recommendation included offering leadership development programs, professional coaching, and negotiation training to enhance career readiness and confidence.
2. Institutional policies: The committee advocated for greater standardization and equity in offer letters, enhanced transparency around compensation, and expanded administrative support for faculty navigating promotion processes, grant applications, and award nominations.
3. Mentorship and sponsorship: The committee proposed that departmental leadership proactively identify and connect faculty with relevant advancement opportunities. Recommendations also emphasized strengthening mentorship systems through improved mentor-mentee pairing and the development of supportive mentorship teams.
4. Monitoring and oversight: To ensure accountability and sustained progress, the committee recommended forming a dedicated advisory group tasked with overseeing the implementation of these initiatives, tracking progress through defined metrics, and making iterative improvements based on outcomes and feedback.

Also classified as highly important, but considered to be more difficult to implement without sufficient resources, were strategies including expanding the mentor base, implementing a departmental midcareer grant, developing sponsorship matching strategies and training, and securing increased administrative support.

Classified as less important but potentially feasible were recommendations including strengthening affinity groups, formalizing mentor assignments to include both near-peer and senior mentors, providing work-life balance-focused faculty development sessions, including financial strategies, and implementing focused strategies to equitably distribute departmental nominations for external awards. Determined to be less important and more difficult, and so considered a lower priority, were initiatives including generic faculty development programs and dependent travel awards.

### 3.4 Summary of Results and Departmental Recommendations

Table 2 provides a summary of final recommendations made by the committee, including action plans and measurement strategies. These were derived after reviewing and revising the recommendations of the consensus review to ensure adherence to current legal and policy requirements. Overall, recommendations that were considered most feasible and important focused on educational efforts (eg, improved academic orientation, coaching), departmental policies (eg, standardization of offers, pay transparency, administrative support for advancement), strengthening mentorship and sponsorship relationships, and creation of an advisory committee to guide implementation and accountability. These will help to support and advance the careers of all faculty.

**Table 2:** Recommendations, strategies, and measurement plans.

Category	Actionable strategy	Measurement plan
Formal academic orientation: improving understanding of academic trajectory and goals	Create academic orientation	Academic orientation in place with monitoring for equitable access
Career trajectory teaching and coaching opportunities	Development of relevant faculty development sessions, peer mentorship program	Implemented faculty development sessions. Monitoring of equity in access/effectiveness of the mentorship program
Leadership and negotiation courses internally	Development of relevant faculty development sessions	Provided faculty development sessions (conflict resolution, negotiation skills, leadership styles, communication, and decision making)
Standardized offers and pay transparency	Department-level offer standardization Departmental report on pay ranges by rank and full-time status	Yearly review by vice chairs of offer letters to ensure consistency and equity Yearly report-out to the faculty with pay ranges
Administrative support for promotion	Standardized administrative support for promotion activities Creating a document or sessions on what is needed at each level for promotion and how to navigate steps Have regular CV reviews to identify what is needed for promotion	Monitor faculty promotion submission and success rates; yearly report-out to faculty
Support to apply for grants/awards	Grants management support has been integrated or increased Administrative support provided for large national award nominations	Monitor faculty grant and award submission and award metrics; yearly report-out to faculty Track and share buy-down numbers, both grant, admin, and self-buy-down Connecting eligible faculty to appropriate local grant mechanisms (eg, for new parents) Track national awards and leadership role numbers
Leadership team connecting faculty to external courses and leadership opportunities	Add to the annual review process	Number of faculty taking courses and course type (topic, internal v. external) Support research training courses for faculty
Mentor pairing and mentorship cohort for junior faculty	Mentorship team development and build out peer mentorship program Regular meetings, topics (eg, self-buy down, mentee/mentorship skills, promotion prep) Development of additional metrics with review from the advisory committee Incentivize mentors for time/effort in departmental metrics	Monitor the number and quality of peer mentorship groups and meetings Faculty perception of peer mentorship program
Advisory committee	Increased opportunity for faculty to provide input on new policies and decisions	Creation and sustainment of an advisory committee Metrics reported to the advisory committee and full faculty

## 4 Limitations

Limitations of this report include that the study was done with the focus on a single department of emergency medicine within an urban academic medical center. This may impact generalizability to other specialties or settings. Bias may have been introduced by the sample of individuals participating in our key informant interviews, which may reflect a subset of faculty more engaged with equity issues. Additionally, we did not explicitly focus on intersectional identities, where barriers, strategies, and approaches may have additional nuance. Methodologically, our literature review and qualitative work were less developed than would be expected for a rigorous qualitative study, but were focused to address the objectives of our project.

## 5 Discussion

This composite assessment of strategies for addressing gender disparities within an academic department of emergency medicine identified several key themes and actionable approaches. Our aim was to develop a framework to guide a comprehensive departmental approach. With a specific approach outlined in Table 2, these strategies focus on the successful recruitment, retention, and advancement of women in academic careers, collectively aiming to build a strong pipeline of women leaders in emergency medicine. Reflecting the broad and interconnected nature of the barriers identified, our findings span multiple domains—ranging from institutional policies to mentorship structures—underscoring the need for multifaceted, multimodal, and sustained interventions.

Among the most quantifiable metrics of gender equity, pay disparities were identified as one of the key areas to address. Prior work has demonstrated that even when salary growth rates are similar between genders, differences in starting salary lead to substantial and compounded differences in earning potential within the first 10 years of employment.^3^ Differences in approaches to negotiation, perceptions while negotiating, and the success of negotiations likely contribute to these differences.^36^^,^^37^ Although targeting negotiation skills is certainly valuable, a more equitable and sustainable solution is the standardization of starting salaries. This approach has been demonstrated to have greater potential impact than equalizing annual salary growth rates.^3^

Many potential solutions that are only available for women may be increasingly challenging to implement. As highlighted by the issue of salary equity, there are many ways in which disparities may be addressed through the development of standardized systems targeting the domains identified in this work and addressing inequities among the entire faculty group. Systemic improvements can help mitigate disparities without relying on demographic-specific programming. This includes ensuring equitable access to administrative support, leadership courses, and career coaching opportunities. During our qualitative interview work with key informants, we found that many women may have less awareness of, or expectations for, such opportunities for growth and advancement. Though we did not hear specific justification or explanation for why this might be during our interviews, prior literature has suggested that entrenched barriers such as organizational culture, under-sponsorship, and informal networking patterns may contribute.^1^^,^^6^^,^^8^^,^^9^ Similarly, a standardized approach to the development of mentorship teams may enable leadership to consider the composition of mentorship for all faculty members, ensuring similar access to mentors who may have more influence and be able to provide greater sponsorship and opportunities for advancement. The set of recommendations provided in Table 2 was developed with current constraints in mind, has been a key guide for strategies underway in our own department, and may serve as a useful starting point for other academic departments to identify strategies that are feasible to implement.

As with all initiatives, measurement of progress and strategies for ensuring both the effectiveness of interventions and the accountability of leadership are critical. Based on this work, our recommendations include concrete plans to address the highest priority recommendations and the development of an advisory committee to ensure ongoing oversight and accountability of this work in our department.

Through a comprehensive literature review, qualitative evaluation, and consensus-building process, this study identified feasible strategies for academic departments to address gender disparities and support the advancement of women in academic and leadership roles. A focus on system-level approaches will promote faculty advancement through standardization across multiple domains, including salary, support, and opportunities. In doing so, departments not only address gender disparity and inclusion challenges but also facilitate the professional growth and promotion of all faculty members.

## Author Contributions

This study was conceived by MES, data collection and analysis were completed by MK and DJ, critical review and evaluation of results were completed by KSZ, DJ, NA, REC, WC, VD, DJE, ELN, PP, ET, and MES; the manuscript was drafted by KSZ with review and editing by MK, DJ, NA, REC, WC, VD, DJE, ELN, PP, ET, and MES. Study supervision was by MES.

## Funding and Support

By *JACEP Open* policy, all authors are required to disclose any and all commercial, financial, and other relationships in any way related to the subject of this article as per ICMJE conflict of interest guidelines (see www.icmje.org). The authors have stated that no such relationships exist.

## Conflict of Interest

The authors declare that they have no known competing financial interests or personal relationships that could have appeared to influence the work reported in this article.
