# Supplementary material for: Organizational and Systems-Based Framework to Guide a Departmental Approach to Academic and Leadership Advancement for Women in Emergency Medicine
Source: J Am Coll Emerg Physicians Open. 2026 Apr 17;7(3):100394. doi: 10.1016/j.acepjo.2026.100394 (PMC13098590; doi:10.1016/j.acepjo.2026.100394)
Supplement: Supplementary Material [file mmc1.doc]

SUPPLEMENTAL MATERIAL to

Organizational and Systems-based Framework to Guide a Departmental Approach to Academic and Leadership Advancement for Women in Emergency Medicine

1. Literature search strategy
2. Literature search results
3. Full roster of committee members
4. List of programs identified by interviewees
5. Literature Search Strategy

The literature search was conducted on July 9, 2024 using the following strategy:

Our research librarian, Jillian Carkin, used keywords related to our main question, “ practices to promote career advancement in women in the field of emergency medicine” to conduct a PubMed search. Two types of searches were performed to identify previously published, relevant articles:

1. The first search was more targeted, using the following specific terms, it yielded 54 results**:**

("Career Mobility"[Mesh] or "career advanc*"[tiab] or "career develop*"[tiab]) **AND**

("Female"[Mesh] or "Physicians, Women"[Mesh] or "woman"[tiab] or "women"[tiab] or "gender disparit*"[tiab] or "gender equity"[tiab] or "gender barrier*"[tiab] or underrepresent*[tiab]) **AND** ("Emergency Medicine"[Mesh] or "emergency medicine"[tiab] or "academic emergency medicine"[tiab])

1. To ensure a more comprehensive overview, a second broader search was conducted using a wider combination of keywords, it yielded 114 results**:**

("Career Mobility"[Mesh] or "career advanc*"[tiab] or "career develop*"[tiab] or "promotion"[tiab] or "innovation"[tiab]) **AND** ("Female"[Mesh] or "Physicians, Women"[Mesh] or "woman"[tiab] or "women"[tiab] or "gender disparit*"[tiab] or "gender equity"[tiab] or "gender barrier*"[tiab] or underrepresent*[tiab]) **AND** ("Emergency Medicine"[Mesh] or "emergency medicine"[tiab] or "academic emergency medicine"[tiab])

1. Literature Search Results

| **1st Author (Year)** | **Title** | **Setting** | **Study Design** | **Key Findings** |
| --- | --- | --- | --- | --- |
| Bryn E Mumma (2017)^1^ | Career Development Awards in Emergency Medicine: Resources and Challenges | Academic ED | Non-intervention (Online cross-sectional surveys of vice chairs for research and research directors) | In this survey study of academic emergency departments, the authors found that low motivation and insufficient mentorship were the most frequent barriers to junior faculty applying for career development awards. Through quantitative analysis it was also found that R-level funded researchers in the department correlates with a larger number of funded career development awards. |
| J Scott Van Epps (2011)^2^ | Early career academic productivity among emergency physicians with R01 grant funding | Non-ED | Non-intervention (data collection and analysis) | In a study of Emergency Physicians with R01 funding from the NIH, the authors found that receiving an R01 grant requires more than a decade of additional research training, publications, and additional smaller grants after completing residency. |
| Corrie E Chumpitaz (2022)^3^ | Career Development in Pediatric Emergency Medicine: What Do We Need? | Pediatric Emergency Medicine | Review of the literature , Survey | The Women in Pediatric Emergency Medicine (PEM) subcommittee of the American Academy of Pediatrics Section on Emergency Medicine conducted a survey of its members to catalog career development domains for women in PEM. From this, they determined that the career development needs of women in PEM consists of a range of personal, teaching, research, administrative, clinical, and service domains. |
| Daniel K Nishijima (2013)^4^ | Description and productivity of emergency medicine researchers receiving K23 or K08 mentored research career development awards | Non- ED   Emergency Medicine Investigators | Non-intervention (cross sectional study) | In a survey of EM researchers who received a K23 or K08 award, researchers found that over 40% of EM K awardees who completed their career development training later obtained federal funding as a principal investigator (PI) or co-PI. Overall, the results show that EM K awardees demonstrate good postaward accomplishments. |
| Moon O Lee (2023)^5^ | Career Advancement Among Women Physicians in Nine Academic Medicine Specialties | Non-ED | Non-intervention (data collection and analysis) | Using rank equity index (REI), the authors of this study found that gender inequity in advancement is evident in academic medicine starting at the assistant professor to associate professor categories, despite the overall proportion of women in the specialty. These results suggest that a common set of barriers to career advancement of women faculty in academic medicine must be communicated in the early stages of one's career. |
| Laksmi Sakura Govindasamy (2022)^6^ | Gender equity in emergency medicine: five years on, where are we headed? | General ED | Non-intervention (literature review?) | In an article exploring the gendered leadership gap in EM, the ACEM (Australiasian College for Emergency Medicine) maintains that broader organizational processes and creating a local evidence-base to support diversity in leadership development remain a priority. |
| Matthew E Purdy (2021)^7^ | Gender differences in publication in emergency medicine journals | General ED | Non-intervention (scoping review) | In a cross-sectional study of published articles in the top three emergency medicine journals, the authors found that males outnumber females in terms of numbers of publications, number of citations, h-index and last authorship. |
| Kirsten Bechtel (2024)^8^ | Pediatric Emergency Medicine and Mentoring: What Women Want | Women in Pediatric Emergency Medicine | Non-intervention (qualitative study: recorded interviews and analysis of data collected) | In a qualitative study of 22 female PEM physicians, four main themes were identified: benefits of mentorship, finding mentors, characteristics of successful mentors, and the impact of mentorship.These findings provide guidance for mentoring programs that address the needs of women in PEM. |
| Cynthia D Morris (2022)^9^ | Outcomes of the National Heart, Lung, and Blood Institute K12 program in emergency care research: 7-year follow up | General ED | non-intervention (electronic survey) | This study describes the outcomes of clinician-scientist scholars in the National Heart Lung and Blood Institute (NHLBI) K12 program in emergency care research across 6 sites in the US. After 7 years of follow up, three quarters of clinician-scientist scholars had obtained CDA or RPG funding and there were no notable differences between sex or clinical training. |
| Gillian Sheppard (2024)^10^ | Perceptions of gender equity in emergency medicine in Canada | General ED | Non-intervention (analysis of previously done survey) | In a secondary data analysis of a survey of Canadian emergency physicians, the authors found that women-identifying physicians were less likely to perceive the there was gender equity in EM in comparison to men-identifying physicians. Additionally, women-identifying physicians agree that there are greater barriers for career advancement and fewer opportunities for leadership, gender wage gap, lack of parental leave policies supporting a return to work, and a lack of education for men to become allies, while men-identifying physicians were less aware of these issues. |
| Judith A Linden (2022)^11^ | Diversity of leadership in academic emergency medicine: Are we making progress? | Academic ED | Non-intervention (cross-sectional observation of disparities among women and URiM) | In a six year cross-sectional observational study, the authors found that there was no change in disparities in women and URiM faculty leadership and academic rank. The results suggest early career inequities as a target for future interventions. |
| Katelyn Moretti (2020)^12^ | Let's Get Personal: Academic Office Displays and Gender | Offices of physicians in varying specialties | General Intervention | In a study of forty-eight physicians' office setup, the authors found that women displayed more personal items than men, however, there was no difference in display of professional items. |
| Linda Regan (2021)^13^ | Geographic Mobility in the emergency medicine residency match and the influence of gender | General ED | Non-intervention (statistical analysis using ArcGIS) | In a study comparing movement between medical school and residency by gender, the authors found that women and men travel similar distances for EM residencies. The majority stayed within geographical proximity to their medical school. |
| Andrea C Fang (2021)^14^ | National Awards and Female Emergency Physicians in the United States: Is the Recognition Gap" Closing? | General ED | Non-intervention (analysis of EM awards given to men vs women) | This study measures the gender distribution of major award winners for 3 main national emergency medicine organizations and compares that to the gender distribution of female faculty in emergency medicine departments. The authors conclude that the gender gap in EM awards has narrowed in the last 20 years but still exists, which shows how bias can compound over time to generate gaps in career advancement. |
| M Clery (2020)^15^ | Federal Funding in Emergency Medicine: Demographics and Perspectives of Awardees | Federal Funding for Emergency Physicians | Non-intervention (retrospective database search and survey analysis) | Based on a retrospective database search and survey analysis, the authors found a trend toward increasing grants awarded to EM investigators over the last five years. They also identified barriers to funding, showing that initiatives geared toward support and mentorship of junior faculty (specifically women and minorities and those less heavily funded) are warranted. |
| Cherri Hobgood (2021)^16^ | Using the Rank Equity Index to measure emergency medicine faculty rank progression | Academic ED | ED Intervention (REI analysis) | Using rank equity index (REI) analysis of EM faculty data, authors found that EM women faculty and faculty of color are not achieving rank parity and are disadvantaged at the first tier of promotion. |
| Imam M Xierali (2020)^17^ | Tenure Trends in Academic Emergency Medicine Departments in U.S. Medical Schools | Academic ED | Statistical Comparisons of Data | By assessing long term trends in tenure status in EM department faculty in US medical schools, the authors found that EM faculty size has increased rapidly in the past 30 years, with the vast majority of growth occuring in nontenured faculty, regardless of sex or URM status. These findings highlight the need to review career development for EM particulary among nontenured faculty. |
| Elle Lett (2023)^18^ | Intersectional Disparities in Emergency Medicine Residents' Performance Assessments by Race, Ethnicity, and Sex | General EM Residents | Retrospective cohort study | This retrospective cohort study found that URM female residents were rated lower than white male residents on Milestone assesments, which may reflect intersectional discrimination in physician competency evaluation. These findings show that elininating sex-specific ethnoracial disparieties in resident assesments may contribute to equitable health care and diversity and representation in the emergency physician workforce. |
| Jennifer S Love (2022)^19^ | WOMen professional development outcome Metrics in Academic Emergency Medicine: Results form the WOMEN TUM Modified Delphi Study | Current and past Female ED chairs and Academy for Women in Academic Emergency Medicine presidents | Delphi Study | This study identified metrics that can be used to assess academic ED's gender equity initiatives and the advisory efforts of a departmental women's professional development groups (PDG). PDG's can use metrics to develop and assess programming, acknowledging that many metrics are the responsibility of the department not the PDG. |
| Stacey Frisch (2024)^20^ | Women's professional development programs for emergency physicians: A scoping review | General ED | Non-intervention: Scoping Review | In a scoping literature review of women's professional development programs for EM physicians, the authors found common program objectives like providing mentors and barriers including lack of funding and support. Overall the finings can inform development of programs that promote gender equity and the advancement of women in EM. |
| Sara M Krzyzaniak (2019)^21^ | What Emergency Medicine Rewards: Is There Implicit Gender Bias in National Awards? | General ED and Academic ED | Non-intervention (Data Analysis) | This study reviewed awards from 5 national organizations over the past 5 years and it showed that in emergency medicine the percentage of women represented in national awards closely mirrors the overall representation of women in emergency medicine. This finding contrasts the documented leadership gap in academic medicine and in EM. |
| Johnlukas Webb (2021)^22^ | First and last authorship by gender in emergency medicine publications- a comparison of 2008 vs 2018 | Women within EM | Retrospective review | In a retrospective review of published original articles during 2008 and 2018, the authors found that female physicians were proportionately represented as first authors in several of the most prominent US based EM journals, even though female physicians make up a disproportionate 28% of the academic workforce. |
| Pooja Agrawal (2019)^23^ | Gender Disparities in Academic Emergency Medicine: Strategies for the Recruitment, Retention, and Promotion of Women | Female academic emergency medicine (EM) physicians | Academic Emergency Medicine Education and Training | This white paper describes recent eveidence demonstrating gender disparities in the EM workforce. The authors make specific recommendations to include individual as well as institutional/systems-level approaches to creating directed programming and initiatives to recruit, support, and promote women at all aspects of the career continuum. |
| Emily M Graham (2021)^24^ | Gender-based Barriers to the Advancement of Women in Academic Emergency Medicine: A Multi-Institutional Survey Study | Women faculty and residents | Multi-institutional electronic survey | In this survey study of women faculty and residents, the authors found that targeted interventions to promote female leadership in academic emergency medicine include coaching on negotiation skills, improved resources and mentorship to support research, and enforcement of safe work environments. Additionally, they found that female emergency physician resiliency is high and not a barrier to career advancement. |
| Kriti Bhatia (2015)^25^ | An Innovative Educational and Mentorship Program for Emergency Medicine Women Residents to Enhance Academic Development and Retention | Women residents | Non-intervention | The authors designed a program to provide accessible mentorship and role models for the trainees at their departmental and instiutional levels. This program is an important step towards encouraging women's pursuit of academic careers, and the authors hope it may be useful to other emergency medicine residencies looking to support their women residents. |
| Dayle Davenpor (2022)^26^ | Faculty Recruitment, Retention, and Representation in Leadership: An Evidence-Based Guide to Best Practices for Diversity, Equity, and Inclusion from the Council of Residency Directors in Emergency Medicine | Faculty in EM | Review of the literature | The authors provide a literature review and offer evidence based guidelines for faculty recruitment, retention, and representation in leadership. These guidelines include targeted recruitment to expand the candidate pool, holistic review of applications, incentivizing stakeholders for success with diversity efforts, establising a culture of inclusivity, promoting faculty development, and evaluating for biases in the promotion and tenure process. |
| Laura Oh (2021)^27^ | Overcoming barriers to promotion for women and underrepresented in medicine faculty in academic emergency medicine | Women and underrepresented minorities in EM | Non-intervention | This article explores potential solutions to addressing inequalities that hinder the advancement and URiM faculty and is intended to complement the recently approved ACEP policy statement also aimed at these barriers. The authors conclude that by publishing a policy statement aimed at these inequalities, ACEP is demonstrating its commitment to a vision of emergency medicine that includes fair advancement and leadership roles for women and URiM emergency medicine physicians. |
| Lois K Lee (2021)^28^ | Addressing gender inequities: Creation of a multi-institutional consortium of women physicians in academic emergency medicine | Women EM faculty | Multicenter Study | This article describes the creationof a cross-institutional consortium focused on career development, academic productivity, and networking and sharing best practices for work-life integration for academic EM women faculty. |
| Mindi Guptill (2018)^29^ | Deciding to lead: a qualitative study of women leaders in emergency medicine | Female EM leaders | Qualitative study using semi-structured interviews | In this qualititative study of female EM leaders, four themes were identified: women leaders made an intentional decision to pursue opportunities to influence emergency medicine, women sought out natural mentors and sponsors to facilitate career development, women leaders intentionally planned their out of work life to support their leadership role, and an important focus for their work was to help others achieve excellence. These findings show leadership in academic medicine is changing to a more democratic horizontally organized management structure which should complement the strengths women bring to the leadership table. |
| Kathleen J Clem (2008)^30^ | Factors enhancing career satisfaction among female emergency physicians | Women emergency physicians in the American College of Emergency Physicians | Survey questionnaire | In this survey study of two thousand five hundred two ACEP female members, the authors found that most of the ACEP female physicians were satisfied with their career choice of emergency medicine. Key factors in this career satisfaction included opportunities for career advancement, fairness in financial compensation, and schedule flexibility. |
| Gabrielle A Jacquet (2014)^31^ | Career paths and practice patterns of global emergency medicine physicians: a cross-sectional survey | Physicians engaged in Global Emergency Medicine | Online survey | In this survey study of 116 attending emergency physicians engaged in GEM, the authors found that female respondents were younger and younger respondents were more likely to have completed advanced GEM training. They found that lack of time and funding were the most common barriers to a career in GEM. |
| Kristi Maso (2022)^32^ | Qualitative description of sexual harassment and discrimination of women in emergency medicine: Giving the numbers a voice | Female EM physicians | An electronic survey | In this qualitative survey study of 1280 female EM physicians, authors found that women in EM experience sexual harassment and discrimination at work by their peers and supervisors. Exploring the themes of these experiences can guide efforts on prevention and intervention. |
| Brielle Stanton Skotnicki (2024)^33^ | Work-Life Integration for Women in Pediatric Emergency Medicine: Themes Identified Through Group Level Assessment | Women physicians in pediatric emergency medicine | Virtual focus group | Virtual focus groups of women PEM physicians found gender inequities, leadership, and balance with family life as major themes affecting their burnout, career satisfaction, and work-life integration. The focus groups also identified actions steps that can be used by individuals and institutions to improve work-life integration for women PEM physicians. |
| Laura E Walker (2024)^34^ | Gender dynamics and academic rank in emergency medicine collaboration networks: A social network and gender propensity analysis | Faculty at two EM departments | Social network analysis | This study used network analysis and gender propensity to identify patterns of collaboration in academic EM. They found no difference in the ways that men and women publish together, however, they did find that individuals with higher academic rank had more importance to the network, regardless of gender. |
| Michelle P Lin (2019)^35^ | Impact of a Women-focused Professional Organization on Academic Retention and Advancement: Perceptions From a Qualitative Study | Physicians | Interviews | This qualitative study of 17 national woman-focused organization leaders found dominant themes of facilitating academic advancement through scholarly productivity, leadership experiences, awards, and promotions; mentorship and sponsorship; peer support and collaborations; reduced professional isolation; and initiatives to address systemic gender inequities and challenges, including strategies to navigate bias, promote pay equity, and advocate for family-friendly workplace policies. |
| Jennifer A Newberry (2019)^36^ | Fostering a Diverse Pool of Global Health Academic Leaders Through Mentorship and Career Path Planning | Global Emergency Medicine Academy faculty | Educational training/development | This paper describes activities launched or hosted at the 2019 SAEM Annual Meeting that leverage GEMA’s national and international network to provide guidance and mentorship in global emergency medicine that may not otherwise be available to trainees and junior faculty. The activities included a mentorship roundtable, a version of speed mentoring that focused on areas such as work-life balance, and the GEMA sponsored panel "Empowering Women through Emergency Care Development in LMICs". |
| Isabella Menchetti (2024)^37^ | Describing a novel, national, vertical mentorship program for women in emergency medicine across Canada | Female EM physicians, Medical students | Survey | This study surveyed 60 participants in a vertical mentorship program. The results showed that the implementation of an innovative, national, vertical mentorship program was largely beneficial for the personal wellbeing and professional development of participants. |
| Tracy E Madsen (2022)^38^ | Institutional solutions addressing disparities in compensation and advancement of emergency medicine physicians: A critical appraisal of gaps and associated recommendations | EM faculty | Interviews | In this qualitative study of 53 faculty leaders across the US, the authors noted diversity in the faculty development and promotion processes across institutions.The four main themes identified were: the need for a directed, structured promotion process; provision of structured mentorship; clarity on requirements for promotion within tracks; and transparency in salary structure. Recommendations were developed to address gaps in structured mentorship and equitable promotion and compensation. |
| Ava E Pierce (2019)^39^ | Advancing Diversity and Inclusion: An Organized Approach Through a Medical Specialty Academy | Academy for Diversity and Inclusion in Emergency Medicine (ADIEM) members | Survey | This survey study shows that the formation of a specialized academy within a national medical has advanced academic accomplishments in diversity, equity, and inclusion in emergency medicine among ADIEM leadership. Involvement of URiM and LGBTQ+ faculty in the academy fostered faculty development, mentoring, and educational scholarship. |
| E Bernstein (1999)^40^ | Sabbatical programs and the status of academic emergency medicine: a survey | The chairs of EM residency programs | Survey | In a survey of 120 EM residency program chairs, the authors found that a sabbatical can be beneficial for individuals and institutions, but EPs have not been able to maximize use of these opportunities. The barriers to participation of EM in sabbatical programs can be overcome with creative strategies and the support of professional academic organizations. |
| G Brown (2016)^41^ | A National Faculty Development Needs Assessment in Emergency Medicine | Academic emergency physicians | Online survey | In a cross-sectional study assesing FD needs among EM educators, authors found that EM faculty report generally high satisfaction with the overall FD they have received. However, only half of EM faculty express satisfaction with education FD specifically. |
| Alexandra Davic (2021)^42^ | Disparity in Gender Representation of Speakers at National Emergency Medical Services Conferences: A Current Assessment and Proposed Path Forward | Emergency Medical Services | Cross-sectional analysis | In a cross-sectional analysis of national conferences for EMS providers, the authors found that gender representation at national EMS conferences in the US is not reflective of the current best estimate of the US EMS workforce. |
| Heather M Prendergast (2019)^43^ | Evaluation of an Enhanced Peer Mentoring Program on Scholarly Productivity and Promotion in Academic Emergency Medicine: A Five-Year Review | EM faculty | Program review | This article evaluates the effectiveness of the EPMP by academic productivity and advancement over 5 years. The authors found that the EPMP was effective in mitigating many traditional mentoring challenges faculty in academia face and was successful in improving academic productivity and advancement. |
| Dave W Lu (2020)^44^ | #MeToo in EM: A Multicenter Survey of Academic Emergency Medicine Faculty on Their Experiences with Gender Discrimination and Sexual Harassment | EM faculty | Cross-sectional survey | In a cross-sectional survey study of EM faculty at six programs, authors found that female EM faculty perceived more gender based discrimination in their workplaces that their male counterparts. Additionally, the majority of female and a quarter of EM faculty experienced unwanted sexual behaviors in their careers. |
| K N Hall (1999)^45^ | Residency-trained emergency physicians: their demographics, practice evolution, and attrition from emergency medicine | Residency-trained emergency physicians | Retrospective cohort study | In a retrospective cohort study of EPs, the authors found that respondants were more likely to stay in EM if they had higher reimbursement, were board certified in EM, or did not train in another speciality or fellowship outside of EM. From this cohort, the attrition from EM pratcice was less than 1% per year. |
| Richard D Gordon (2020)^46^ | Evaluating the Diversity of Emergency Medicine Foundation (EMF) Grant Recipients in the Last Decade | Emergency Medicine Foundation (EMF) grant awardees | Survey | In a survey study of EMF grant recipients from the last 10 years, authors found that there was a considerable lack of diversity. The most common barriers perceived by this cohort were medical speciality, gender, and age. |
| Esther K Choo (2016)^47^ | The Development of Best Practice Recommendations to Support the Hiring, Recruitment, and Advancement of Women Physicians in Emergency Medicine | Female EM faculty | Focus groups | A working group consisting of leadership of two EM women's organizations used a consensus building process to develop recommendations for organizations to implement to create a workplace environment supportive of women that were deemed acceptable and feasible. This process can serve as a model for other specialities to develop clear organization -evel practices aimed at supporting women physicians. |
| Molly Allen (2024)^48^ | Women at the top: a qualitative study of women in leadership positions in emergency medicine in Canada | Women emergency medicine physicians | Qualitative study | In a qualitative study of 20 women EPs in Canada, participants emphasized the importance of mentorship, sponsorship, applying for leadership positions early, networking, and empowering younger generations of women to become leaders. Participants perceived gender expectations and traditional gender roles as having a negative impact on career leadership success. |
| Gloria J Kuhn (2008)^49^ | Recommendations from the Society for Academic Emergency Medicine (SAEM) Taskforce on women in academic emergency medicine | Women in EM | Focus groups | The SAEM created a taskforce to study issues pertaining to women in academic EM. The taskforce and authors reviewed literature to recommend practical actions at multiple levels, including leadership of national EM organizations, medical school deans, department chairs, and women faculty members. |
| Justin Morgenstern (2022)^50^ | Hot Off the Press: Addressing gender inequities: Creation of a multi-institutional consortium of women physicians in academic emergency medicine | Women physicians in academic emergency medicine | Program development | This article describes the creation of a consortium of female faculty in emergency medicine that developed events based on needs assesments and literature reviews, and they developed systems for information sharing among hospitals. This article discusses the short term success and challenges and provides advice for others interested in developing a similar program in four key domains: leadership, finances, communications, and curriculum development. |
| Jennifer S Love (2024)^51^ | A Decade in Review: Trends in Female Authorship in Peer-Reviewed Toxicology Journals | Female medical toxicology fellows and medical toxicologists | Retrospective review of all non-abstract publication | In a retrospective review of non-abstract publications in two medical toxicology journals, the authors found that the frequency of female authorship in the first author position has grown over the last decade. They also found that this is associated with increasing female representation in medical toxicology and specific manuscript subtypes, specifically research manuscripts. |
| Emily M Graham (2022)^52^ | This Article Corrects: "Gender-based Barriers in the Advancement of Women Leaders in Emergency Medicine: A Multi-institutional Qualitative Study" | General ED | Quantitative Survey | In a multi-institutional survey of women faculty and residents, authors found that female emergency physician resiliency is high and is not a barrier to career advancement. They also suggested targeted interventions to promote female leadership in academic emergency medicine include coaching on negotiation skills, improved resources and mentorship to support research, and enforcement of safe work environments. |
| Luke Phillips (2016)^53^ | A call for ACEM to act on gender inequity in our training programme: A male perspective | Australian College of Emergency Medicine | Opinion / Editorial | This op-ed details the bullying, harrasment, physical violence and verbal abuse from patients, and limitations to parental leave that emergency physicians, especially women, face. It calls on the ACEM to advocate for its trainees and adopt measures like strong mentorship and flexibility in training to close the gender gap and expand diversity in emergency medicine. |
| V T Chande (2001)^54^ | Practitioners of pediatric emergency medicine: a 5-year longitudinal study | Non-ED (American Board of Pediatrics and the American Board of Emergency Medicine) | Prospective Cohort Study | In this prospective cohort study of 232 PEM sub-board certified physicians, the authors found that the priorities of the cohort changed as the physicians grew older. It showed that lifestyle issues must be taken into consideration to ensure longevity in the subspeciality. |
| Cameron J Gettel (2023)^55^ | Emergency medicine physician workforce attrition differences by age and gender | Male and female emergency physicians | Cross-sectional analysis/ multivariate logistic regression model | In a cross-sectional analysis of EPs reimbursed by Medicare, the authors found that female physicians exhibited attrition from the EM workforce at an age approximately 12 years younger than male physicians. The data identifyed in this article show widespread disparities regarding EM workforce attrition. |

1. Committee Members:

Nour Al Jalbout, MD, Alice Bukhman, MD, MPH, Rebecca E. Cash, PhD, Wee-Jhong Chua, MD, Valerie Dobiesz, MD, MPH, Daniel J. Egan, MD, MBA, Catalina Gonzalez Marques, MD, MPH, Luke Messac, MD, Erica Nelson, MD, MPHIL, MAS, Phuong Pham, PhD, MPH, Margaret Samuels-Kalow, MD, MPhil, MSHP, Elizabeth Temin, MD, MPH, Sara Tuttle-Lane, MD, and Kori S. Zachrison, MD, MSC with staff support from Dara L. James, PhD, MS and Mariam Kapanadze, MPH

1. Specific Programs Suggested by the Interviewees

- Local
- Anne Klibanski Visiting Scholars Award (Mass General Brigham specific)
- Office for Women’s Careers (OWC at Massachusetts General Hospital)
- Brigham Physician Leadership Program
- Brigham Women Innovators Launch
- External
  - Executive Leadership in Academic Medicine (ELAM)
  - American Association for Women in Radiology

Supplement References

1. Mumma BE, Chang AM, Kea B, Ranney ML. Career Development Awards in Emergency Medicine: Resources and Challenges. *Acad Emerg Med*. 2017;24(7):855-863. doi:10.1111/ACEM.13189

2. Van Epps JS, Younger JG. Early career academic productivity among emergency physicians with R01 grant funding. *Academic Emergency Medicine*. 2011;18(7):759-762. doi:10.1111/J.1553-2712.2011.01118.X,

3. Chumpitazi CE, Allister L, Cho C, et al. Career Development in Pediatric Emergency Medicine: What Do We Need? *Pediatr Emerg Care*. 2022;38(9):E1552-E1556. doi:10.1097/PEC.0000000000002720

4. Nishijima DK, Yadav K, May L, Kraynov L, Mark Courtney D. Description and productivity of emergency medicine researchers receiving K23 or K08 mentored research career development awards. *Academic Emergency Medicine*. 2013;20(6):611-617. doi:10.1111/ACEM.12152,

5. Lee MO, Flores B, Fassiotto M, Hobgood C. Career Advancement Among Women Physicians in Nine Academic Medicine Specialties. *J Womens Health (Larchmt)*. 2023;32(10):1073-1079. doi:10.1089/JWH.2022.0464

6. Govindasamy LS, Terziovski M, Wheeler M, Rixon A, Wilson S. Gender equity in emergency medicine: Five years on, where are we headed? *Emerg Med Australas*. 2022;34(2):288-290. doi:10.1111/1742-6723.13910

7. Purdy ME, Zmuda BN, Owens AM, et al. Gender differences in publication in emergency medicine journals. *American Journal of Emergency Medicine*. 2021;49:338-342. doi:10.1016/j.ajem.2021.06.039

8. Bechtel K, Langhan ML, Levine D, Hanson J. Pediatric Emergency Medicine and Mentoring: What Women Want. *Pediatr Emerg Care*. 2024;40(6):449-453. doi:10.1097/PEC.0000000000003192,

9. Morris CD, Cook JNB, Lin A, et al. Outcomes of the National Heart, Lung, and Blood Institute K12 program in emergency care research: 7-year follow-up. *Acad Emerg Med*. 2022;29(10):1197-1204. doi:10.1111/ACEM.14563

10. Sheppard G, McIlveen-Brown E, Jacques Q, et al. Perceptions of gender equity in emergency medicine in Canada. *Canadian Journal of Emergency Medicine*. 2024;26(4):271-279. doi:10.1007/S43678-024-00665-9,

11. Linden JA, Baird J, Madsen TE, et al. Diversity of leadership in academic emergency medicine: Are we making progress? *Am J Emerg Med*. 2022;57:6-13. doi:10.1016/J.AJEM.2022.04.009

12. Moretti K, Musits A, McGregor A, Aluisio A. Let’s Get Personal: Academic Office Displays and Gender. *Permanente Journal*. 2020;24(3). doi:10.7812/TPP/19.237,

13. Regan L, Gisondi MA, Branzetti J, et al. Geographic mobility in the emergency medicine residency match and the influence of gender. *AEM Educ Train*. 2021;5(4). doi:10.1002/AET2.10706

14. Fang AC, Chekijian SA, Zeidan AJ, Choo EK, Sethuraman KN. National Awards and Female Emergency Physicians in the United States: Is the “Recognition Gap” Closing? *Journal of Emergency Medicine*. 2021;61(5):540-549. doi:10.1016/j.jemermed.2021.07.009

15. Clery MJ, Dworkis DA, Sonuyi T, Khaldun JS, Abir M. Federal Funding in Emergency Medicine: Demographics and Perspectives of Awardees. *West J Emerg Med*. 2020;21(2):291-294. doi:10.5811/WESTJEM.2019.12.45249

16. Hobgood C, Fassiotto M. Using the Rank Equity Index to measure emergency medicine faculty rank progression. *Acad Emerg Med*. 2021;28(9):966-973. doi:10.1111/ACEM.14268

17. Xierali IM, Nivet MA. Tenure Trends in Academic Emergency Medicine Departments in U.S. Medical Schools. *AEM Educ Train*. 2020;4(3):202-211. doi:10.1002/AET2.10452

18. Lett E, Tran NK, Nweke N, et al. Intersectional Disparities in Emergency Medicine Residents’ Performance Assessments by Race, Ethnicity, and Sex. *JAMA Netw Open*. 2023;6(9):E2330847. doi:10.1001/JAMANETWORKOPEN.2023.30847,

19. Love JS, Zeidan AJ, Khatri UG, Samuels-Kalow ME, Mills AM, Hsu CH. WOMen profEssioNal developmenT oUtcome Metrics in Academic Emergency Medicine: Results from the WOMENTUM Modified Delphi Study. *West J Emerg Med*. 2022;23(5):660-671. doi:10.5811/WESTJEM.2022.6.56608

20. Frisch S, Desai R, Chung AS, Love JS, Adair White BA. Women’s professional development programs for emergency physicians: A scoping review. *AEM Educ Train*. 2024;8(2). doi:10.1002/AET2.10971

21. Krzyzaniak SM, Gottlieb M, Parsons M, Rocca N, Chan TM. What Emergency Medicine Rewards: Is There Implicit Gender Bias in National Awards? *Ann Emerg Med*. 2019;74(6):753-758. doi:10.1016/j.annemergmed.2019.04.022

22. Webb J, Cambron J, Xu KT, Simmons M, Richman P. First and last authorship by gender in emergency medicine publications- a comparison of 2008 vs. 2018. *American Journal of Emergency Medicine*. 2021;46:445-448. doi:10.1016/j.ajem.2020.10.045

23. Agrawal P, Madsen TE, Lall M, Zeidan A. Gender Disparities in Academic Emergency Medicine: Strategies for the Recruitment, Retention, and Promotion of Women. *AEM Educ Train*. 2019;4(Suppl 1):S67-S74. doi:10.1002/AET2.10414

24. Graham EM, Ferrel MN, Wells KM, et al. Gender-based Barriers to the Advancement of Women in Academic Emergency Medicine: A Multi-institutional Qualitative Study. *Western Journal of Emergency Medicine*. 2021;22(6):1355. doi:10.5811/WESTJEM.2021.7.52826

25. Bhatia K, Takayesu JK, Arbelaez C, Peak D, Nadel ES. An Innovative Educational and Mentorship Program for Emergency Medicine Women Residents to Enhance Academic Development and Retention. *CJEM*. 2015;17(6):685-688. doi:10.1017/CEM.2015.17

26. Davenport D, Alvarez A, Natesan S, et al. Faculty Recruitment, Retention, and Representation in Leadership: An Evidence-Based Guide to Best Practices for Diversity, Equity, and Inclusion from the Council of Residency Directors in Emergency Medicine. *West J Emerg Med*. 2022;23(1):62-71. doi:10.5811/WESTJEM.2021.8.53754

27. Oh L, Linden JA, Zeidan A, et al. Overcoming barriers to promotion for women and underrepresented in medicine faculty in academic emergency medicine. *JACEP Open*. 2021;2(6). doi:10.1002/emp2.12552

28. Lee LK, Platz E, Klig J, et al. Addressing gender inequities: Creation of a multi-institutional consortium of women physicians in academic emergency medicine. *Acad Emerg Med*. 2021;28(12):1358-1367. doi:10.1111/ACEM.14361

29. Guptill M, Reibling ET, Clem K. Deciding to lead: A qualitative study of women leaders in emergency medicine. *Int J Emerg Med*. 2018;11(1). doi:10.1186/S12245-018-0206-7,

30. Clem KJ, Promes SB, Glickman SW, et al. Factors Enhancing Career Satisfaction Among Female Emergency Physicians. *Ann Emerg Med*. 2008;51(6). doi:10.1016/j.annemergmed.2008.01.011

31. Jacquet GA, Hansoti B, Levine AC, Martin IBK. Career paths and practice patterns of global emergency medicine physicians: A cross-sectional survey. *Journal of Emergency Medicine*. 2014;47(3):348-354. doi:10.1016/j.jemermed.2014.01.029

32. Maso K, Theobald JL. Qualitative description of sexual harassment and discrimination of women in emergency medicine: Giving the numbers a voice. *AEM Educ Train*. 2022;6(2). doi:10.1002/AET2.10727,

33. Skotnicki BS, Wilson PM, Kazmerski TM, et al. Work-Life Integration for Women in Pediatric Emergency Medicine: Themes Identified Through Group Level Assessment. *Pediatr Emerg Care*. 2024;40(1):71-75. doi:10.1097/PEC.0000000000003106

34. Walker LE, Heaton HA, Kohler K. Gender dynamics and academic rank in emergency medicine collaboration networks: A social network and gender propensity analysis. *American Journal of Emergency Medicine*. 2024;83:40-46. doi:10.1016/j.ajem.2024.06.037

35. Lin MP, Lall MD, Samuels-Kalow M, et al. Impact of a Women-focused Professional Organization on Academic Retention and Advancement: Perceptions From a Qualitative Study. *Academic Emergency Medicine*. 2019;26(3):303-316. doi:10.1111/ACEM.13699,

36. Newberry JA, Patel S, Kayden S, O’Laughlin KN, Cioè‐Peña E, Strehlow MC. Fostering a Diverse Pool of Global Health Academic Leaders Through Mentorship and Career Path Planning. *AEM Educ Train*. 2019;4(Suppl 1). doi:10.1002/AET2.10403

37. Menchetti I, Pham C. Describing a novel, national, vertical mentorship program for women in emergency medicine across Canada. *CJEM*. 2024;26(6):409-412. doi:10.1007/S43678-024-00715-2

38. Madsen TE, Heron S, Lall MD, et al. Institutional solutions addressing disparities in compensation and advancement of emergency medicine physicians: A critical appraisal of gaps and associated recommendations. *Academic Emergency Medicine*. 2022;29(6):710-718. doi:10.1111/ACEM.14452,

39. Pierce AE, Moreno-Walton L, Boatright D, et al. Advancing Diversity and Inclusion: An Organized Approach Through a Medical Specialty Academy. *AEM Educ Train*. 2019;4(Suppl 1):S40-S46. doi:10.1002/AET2.10427

40. Bernstein E, James T, Bernstein J. Sabbatical programs and the status of academic emergency medicine: a survey. *Acad Emerg Med*. 1999;6(9):932-938. doi:10.1111/J.1553-2712.1999.TB01244.X

41. Brown GM, Lang E, Patel K, et al. A National Faculty Development Needs Assessment in Emergency Medicine. *CJEM*. 2016;18(3):161-182. doi:10.1017/CEM.2015.77

42. Davic A, Carey E, Lambert E, et al. Disparity in Gender Representation of Speakers at National Emergency Medical Services Conferences: A Current Assessment and Proposed Path Forward. *Prehosp Disaster Med*. 2021;36(4):445-449. doi:10.1017/S1049023X21000571

43. Prendergast HM, Heinert SW, Erickson TB, Thompson TM, Vanden Hoek TL. Evaluation of an Enhanced Peer Mentoring Program on Scholarly Productivity and Promotion in Academic Emergency Medicine: A Five-Year Review. *J Natl Med Assoc*. 2019;111(6):600-605. doi:10.1016/j.jnma.2019.07.001

44. Lu DW, Lall MD, Mitzman J, et al. #MeToo in EM: A Multicenter Survey of Academic Emergency Medicine Faculty on Their Experiences with Gender Discrimination and Sexual Harassment. *Western Journal of Emergency Medicine*. 2020;21(2):252. doi:10.5811/WESTJEM.2019.11.44592

45. Hall KN, Wakeman MA. Residency-trained emergency physicians: Their demographics, practice evolution, and attrition from emergency medicine. *Journal of Emergency Medicine*. 1999;17(1):7-15. doi:10.1016/S0736-4679(98)00119-X

46. Gordon RD, Kwon NS, Levy PD, Madsen TE, Greenberg MR. Evaluating the Diversity of Emergency Medicine Foundation (EMF) Grant Recipients in the Last Decade. *West J Emerg Med*. 2020;21(3):595-599. doi:10.5811/WESTJEM.2020.2.46497

47. Choo EK, Kass D, Westergaard M, et al. The Development of Best Practice Recommendations to Support the Hiring, Recruitment, and Advancement of Women Physicians in Emergency Medicine. *Acad Emerg Med*. 2016;23(11):1203-1209. doi:10.1111/ACEM.13028

48. Allen M, Lazor J, Nirmalanathan K, Nowacki A. Women at the top: a qualitative study of women in leadership positions in emergency medicine in Canada. *Canadian Journal of Emergency Medicine*. 2024;26(11):819-827. doi:10.1007/S43678-024-00751-Y,

49. Kuhn GJ, Abbuhl SB, Clem KJ. Recommendations from the Society for Academic Emergency Medicine (SAEM) taskforce on women in academic emergency medicine. *Academic Emergency Medicine*. 2008;15(8):762-767. doi:10.1111/J.1553-2712.2008.00190.X,

50. Morgenstern J, Challen K, Bond C, Milne WK. Hot Off the Press: Addressing gender inequities: Creation of a multi-institutional consortium of women physicians in academic emergency medicine. *Acad Emerg Med*. 2022;29(2):238-240. doi:10.1111/ACEM.14445

51. Love JS, Loo GT, Murphy L, et al. A Decade in Review: Trends in Female Authorship in Peer-Reviewed Toxicology Journals. *J Med Toxicol*. 2024;20(1):22-30. doi:10.1007/S13181-023-00975-X

52. Graham EM, Ferrel MN, Wells KM, et al. This Article Corrects: “Gender-based Barriers in the Advancement of Women Leaders in Emergency Medicine: A Multi-institutional Qualitative Study.” *West J Emerg Med*. 2022;23(2):290. doi:10.5811/WESTJEM.2022.2.56587

53. Phillips L. A call for ACEM to act on gender inequity in our training programme: A male perspective. *EMA - Emergency Medicine Australasia*. 2016;28(3):349-350. doi:10.1111/1742-6723.12598,

54. Chande VT, Krug SE. Practitioners of pediatric emergency medicine: A 5-year longitudinal study. *Pediatr Emerg Care*. 2001;17(4):237-239. doi:10.1097/00006565-200108000-00002,

55. Gettel CJ, Courtney DM, Agrawal P, et al. Emergency medicine physician workforce attrition differences by age and gender. *Acad Emerg Med*. 2023;30(11):1092-1100. doi:10.1111/ACEM.14764
